# The Relationship between Coaching Behavior and Athlete Burnout: Mediating Effects of Communication and the Coach–Athlete Relationship

**DOI:** 10.3390/ijerph17228618

**Published:** 2020-11-20

**Authors:** Hunhyuk Choi, Yunduk Jeong, Suk-Kyu Kim

**Affiliations:** 1 Department of Physical Education, College of Education, Kangwon National University, 1 Kanwondaehak-gil, Chuncheon-si, Gangwon-do 24341, Korea; hchoi20@Kangwon.ac.kr; 2 College of General Education, Kookmin University, 77, Jeongneung-ro, Seongbuk-gu, Seoul 02707, Korea; jeongyunduk@kookmin.ac.kr; 3 Department of Sport Science, College of Humanities, Dongguk University Gyeongju, 123 Dongdae-ro, Gyeongju-si, Gyeongsangbuk-do 38066, Korea

**Keywords:** coaching behavior, autonomy-support, controlling, communication, coach–athlete relationship, burnout

## Abstract

The purpose of this study was to investigate the relationships between perceived coaching behavior (autonomy-supportive and controlling), communication, coach–athlete relationship, and athlete burnout. The study participants comprised 347 Korean active collegiate athletes from 10 sports. The results of the final model indicated that autonomy-supportive coaching was positively related to communication, whereas controlling coaching was negatively related to communication. Communication was positively related to coach–athlete relationship and was negatively related to athlete burnout. Autonomy-supportive coaching was significantly related to both the coach–athlete relationship (positively) and athlete burnout (negatively), whereas controlling coaching was only related to athlete burnout (positively). Coach–athlete relationship was negatively related to athlete burnout. Significant indirect effects were observed. The bootstrapping results indicated that the relationship between autonomy-supportive and athlete burnout was mediated by team communication and the coach–athlete relationship. The study findings enhance our current understanding of the relationships between perceived coaching behavior and athlete burnout and shed light on the important roles of team communication and the coach–athlete relationship in the relationship.

## 1. Introduction

Athletes can experience severe or excessive stress in a sports environment. In recognition of the importance of athlete burnout in sports situations, [1] carried out a specific reconceptualization of burnout and developed the Athlete Burnout Questionnaire (ABQ) to measure three dimensions of burnout in sports situations: emotional and physical exhaustion, sport devaluation, and a reduced sense of accomplishment. The development of the ABQ has contributed significantly to the development of research on burnout among athletes. Conflicts between coaches and athletes and between team members or colleagues may be manifested as symptoms, such as poor athletic performance, dropout [2], and athlete burnout [1,3]. In particular, the levels of control and autonomy support in coaching behaviors have also been studied from a motivational perspective to predict athlete burnout through psychological needs and motivational regulations [4]. Davis et al. [5] emphasized the important role social factors play in preventing athlete burnout, and Quested and Duda [6] reported that athletes are likely to experience higher levels of burnout if coaches show strict or controlling coaching behaviors or do not provide autonomy support.

One of the main reasons why communication is attracting attention in sports is that athletes’ perception of the communication methods of their coaches affects the atmosphere of practice and training, participation, and athletic performance. Communication is a potential mechanism through which the quality of the coach–athlete relationship may affect interpersonal relationships. Gilbert [7] stated that communication is an effective coaching strategy for building and maintaining coach–athlete relationships. In particular, Carron and Hausenblas [8] argued that effective communication within the team is essential for developing and maintaining the team structure. Furthermore, Joweet and Wylleman [9] emphasized the importance of communication between the coach and athletes in sports environments to prevent athlete burnout.

Overall, as increasing attention has been paid to reducing or preventing athlete burnout in various sports (individual and team sports), research has attempted to identify the factors affecting burnout. As mentioned above, coaching behaviors and coach–athlete relationships have a positive effect on the reduction or prevention of athlete burnout. On the other hand, there are no reports on the mediating effects of communication and coach–athlete relationship on the relationship between coaching behavior and athlete burnout. Therefore, this study examined the mediating effects of communication and coach–athlete relationship on the relationship between coaching behavior and athlete burnout.

## 2. Literature Review

### 2.1. Athlete Burnout

Conflict between a coach and athlete or between teammates can manifest as a decrease in athletic performance, dropping out [2], or symptoms of burnout [1,3]. According to Smith [10], there are various reasons why athletes terminate their athletic careers, but burnout is one of the main causes. So far, a number of studies have been conducted from the perspective of individual characteristics and socio-psychological factors in order to prevent athlete burnout. Athlete burnout has mainly been explained from two aspects: the process toward, and the state of, burnout. In particular, in the cognitive–affective stress model proposed by Smith [10], burnout is hypothesized as developing in a four-stage process during which stress and burnout evolve in parallel. The four stages are the situation, cognitive appraisal, physiological responses, and behavioral responses or a decrease in coping ability, such as decreased performance. In addition, Silva [11] developed a conceptual model of the training stress syndrome, focusing on physical and training factors while recognizing the importance of psychological aspects. While these models applied the concept of athlete burnout mainly in terms of stress, the investment model proposed by Schmidt and Stein [12] suggested that athletes are at risk of burning out if they continue to participate in sports because they cannot give up careers in which they have invested a great deal (e.g., time, emotion, friendship, and dealing with pressure from parents and the coach), and because they have few alternatives [13]. On the other hand, in the commitment model proposed by Raedeke [14], under the assumption that “not all athletes experiencing stress will experience burnout,” limitations on the stress perspective were mentioned, and commitment was presented as an important factor in the process of developing burnout. In other words, most of the reasons for individuals’ involvement in an interpersonal relationship or an occupation were explained from a commitment perspective [15].

Most of the studies on athlete burnout in Korea have attempted to explain the process of athlete burnout based on a structure composed of three dimensions suggested by previous studies [1,4,6,16], and they were mostly based on the cognitive–affective stress model. Recently, some studies on athlete burnout were conducted from the perspective of motivation [17,18], and they reported that control and the autonomy-supportive level in coaching behavior can predict athlete burnout, directly or indirectly, through psychological needs and motivation regulation [4]. So far, some prior studies based on a leadership perspective or on the self-determination theory have reported that coaches’ strict or controlling behaviors, failure to provide autonomy-supportive coaching [6], and a low level of social support from coaches are associated with a higher risk for, or a higher level of, athlete burnout [1].

### 2.2. Coaching Style

Several previous studies based on the self-determination theory have consistently demonstrated that autonomy-supportive coaching behaviors by leaders in the sports field (coaches, head coaches, or managers) are more effective than behaviors that are controlling. The qualitative studies of athlete burnout by Cresswell and Eklund [19] and Gustafsson et al. [20] emphasized the fact that dissatisfaction in the relationship between coaches and athletes, such as conflicts, unsatisfactory communications, and a lack of empathy in coaches are associated with athlete burnout. Negative social interactions especially, such as unsolicited advice or intervention, not providing help when an athlete asks for it, and disregard for individuals who are prominently observed in sports, were found to be strong predictors of athlete burnout [21]. These study results demonstrated that coaches’ autonomy-supportive behaviors have a more positive impact in the field of sports than coaching that is controlling. Consistent with the findings, Cheon and Reeve [22] proved positive effects from the autonomy-supportive method by providing teachers who participated in an autonomy-supportive teacher training program with education on methods of communicating with students in a less controlling and more autonomy-supportive way. The study also reported that a coach’s controlling behavior causes conflict between the coach and the athletes, and negatively affects athletes’ motivation, achievement, and performance. These empirical results provide evidence for the argument that coaches should pursue and utilize methods that empathize with, and support, the athletes. Seong [23] claimed that coaches’ behaviors play a decisive role in helping individuals or groups reach their goals. Although many prior studies on coaching behavior have so far focused on identifying the characteristics and types of coaching behaviors and the antecedent variables that affect coaching behaviors, it is now necessary to pay attention to studies that elucidate the results or effects of coaching behavior.

### 2.3. Communication and Coach–Athlete Relationship

Coaches, including head coaches and managers, have a great influence on the behavior of athletes as socially influential people or through smooth interactions with athletes. Interpersonal relationships between coaches and athletes are a central part of the coaching process. This type of coaching is an important element for high performance and plays a key role in ensuring athletes’ continued success. Recently, as the importance of coaching in the field of sports has increased, many researchers have paid a great deal of attention to coaching behavior, and related research has also been increasing. In particular, Eccles and Tran [24] reported that effective communication within a team is an essential element for the development and maintenance of team structure, and Joweet and Wylleman [9] emphasized the importance in a sport environment of communication between coaches and their athletes in order to proactively prevent athlete burnout. Jung, Lim and Choi [25] reported that cooperative relationships with leaders and teammates in sport situations have a positive effect on team performance, but a disagreement among team members has a negative impact on both individual and team performance [26]. In addition, one of the most important reasons why communication is receiving increased attention as an important factor in the field of sports is that the atmosphere of practice and training, participation, and performance are affected by how athletes perceive the coach’s method of communication. According to Bippus, Kearney, Plax, and Brooks [27], athletes who engage in mutual communication with their coaches in other situations as well as during practice, training, and sports events are more likely to consider their coaches trustworthy and reliable, because they perceive coaches as genuine or accessible. In addition, it has been reported that if coaches respond positively to athletes’ opinions and behaviors, it increases cognitive learning effects, and as a result, communication with coaches is more actively utilized [28,29,30]. On the other hand, Loughead and Carron [31] stated that unidirectional communication from a coach’s unilateral direction and through coercion is less efficient than bidirectional communication in the relationship between coaches and athletes, and it has a negative impact, especially on athletic performance. These previous studies demonstrated that if athletes maintain a positive relationship and communicate effectively with their coaches, it can lead to effective results from various aspects.

As described above, if athletes are given autonomy, which allows them to think freely in their interactions with the coach, and if the coach listens attentively to them, makes a positive impression as a trustworthy person, and adjusts more flexibly to communications with athletes [32], it will not only help to maintain a good relationship between athletes and their coaches but also reduce or prevent athlete burnout. Previous studies [33,34] found that if smooth communications between the leader and athletes is possible, athletes are more likely to strive to achieve their goals, and they found that open and free communication between athletes and their coaches in this process is associated with a lower level of athlete burnout. Based on the above findings, socio-psychological variables are expected to play some role in reducing or preventing athlete burnout. Therefore, this study investigates whether socio-psychological variables have a direct positive effect on preventing or reducing athlete burnout, and it looks at what roles communication and the coach–athlete relationship play in the relationship between coaching behavior and burnout.

If positive roles from communication and the coach–athlete relationship are confirmed in this study, research on the importance of interpersonal aspects can be further intensified. In addition, it is expected that the results of this study can be used as positive data for the development of education programs for sport leaders, along with maintenance strategies for the improvement of interpersonal relationships and the formation of positive coach–athlete relationships through positive roles of postulated mediator variables.

### 2.4. Relationship among Coaching Behavior, Communication, and Coach–Athlete Relationship and Athlete Burnout

The purpose of this study is to identify the relationship among coaching behavior (autonomy-support coaching and controlling coaching), communication, and coach–athlete relationship and athlete burnout. First, previous studies [21,22] indicated the autonomy-support coaching is more effective to reduce the athlete burnout than controlling coaching. In addition, autonomy-support coaching has been identified as positively affecting communication and social relationship [25,35]. Second, prior studies found [15,36,37] that communication has a positive effect on athlete burnout. Communication has also been identified as positively affecting on coach–athlete relationship [24,27,28,29,30,31]. Third, previous studies [19,20,21,25] found that coach–athlete relationship is inversely related to athlete burnout. Fourth, prior studies [19,20,21,22,24,25,27,28,29,30,31,32,33,34] suggested the mediating effect of communication and coach–athlete relationship between coaching behavior and athlete burnout. Based on these prior findings, we propose the following hypotheses:

**Hypothesis** **1.** *Autonomy-support coaching will have a positive effect on athlete burnout.*

**Hypothesis** **2.** *Autonomy-support coaching will have a positive effect on communication.*

**Hypothesis** **3.** *Autonomy-support coaching will have a positive effect on coach–athlete relationship.*

**Hypothesis** **4.** *Controlling coaching will have a negative effect on athlete burnout.*

**Hypothesis** **5.** *Controlling coaching will have a negative effect on communication.*

**Hypothesis** **6.** *Controlling coaching will have a negative effect on coach–athlete relationship.*

**Hypothesis** **7.** *Communication will have a positive effect on athlete burnout.*

**Hypothesis** **8.** *Communication will have a positive effect on coach–athlete relationship.*

**Hypothesis** **9.** *Coach–athlete relation will have a positive effect on athlete burnout.*

**Hypothesis** **10.** *Autonomy-supportive coaching will have an indirect effect on coach–athlete relationship mediated by communication.*

**Hypothesis** **11.** *Autonomy-supportive coaching will have an indirect effect on athlete burnout mediated by communication and coach–athlete relationship.*

**Hypothesis** **12.** *Controlling coaching will have an indirect effect on coach–athlete relationship mediated by communication.*

**Hypothesis** **13.** *Controlling coaching will have an indirect effect on athlete burnout mediated by communication and coach–athlete relationship.*

## 3. Methods

### 3.1. Participants

This study used convenience sampling to select participants. A total of 400 surveys were distributed to 5 different colleges in Kangwon, South Korea. Among the collected data, questionnaires with insincere and incomplete responses (e.g., biased responses, missing data, repeated patterns, etc.) were excluded from the analysis, and only valid samples (347 in total) were used. Of the participants, 87.0% were male, 13.0% were female, mean age was 21.6. Table 1 presents the participants’ demographic information.

### 3.2. Procedure

Before conducting the survey, the researcher completed an online educational course on the Bioethics and Safety Act conducted by the IRB of the institution to which the researcher belongs, taking into consideration research ethics. Data collection was conducted after requesting the cooperation of participants and by obtaining consent from the person in charge (coach, head coach, or manager) of the college sports team by sending information about the contents and procedure of the study by e-mail in order to fully explain the purpose.

The specific methods and procedures for data collection were as follows. First, in order to collect research data, the researcher and two assistants personally visited each university to explain the study’s purpose, the method for completing the questionnaire, and any precautions for the athletes. Second, informed consent was obtained from participants, who had to voluntarily agree to participate; to minimize insincere responses and to avoid missing data, participants were assured that the results would only be used as basic data for this study, and not for other purposes. Third, participants were informed that all individual characteristics and questionnaire data about the participants would be anonymized to ensure confidentiality, and they were provided with a sufficient explanation about the contents of the information provided so they could clearly understand the procedure during the survey process. It took about 10 min for each participant to complete the questionnaire, which was collected immediately after being completed.

### 3.3. Instruments

The survey consisted of six sections, including autonomy-supportive behavior, controlling behavior, communication, coach–athlete relationship, athlete burnout, and demographic information. To measure the perceived autonomy-supportive coaching behavior among college athletes, a Korean version of the Sport Climate Questionnaire (SCQ) developed by Deci [38] was used. This instrument is a scale to assess perceived autonomy support, and the Korean version of the SCQ was validated by Kim and Park [39]. It is a six-item short-form questionnaire and includes statements such as “the coach gives me choices and opportunities” and “the coach generally recognizes me as an athlete.” Each respondent is asked to rate each item on a seven-point Likert scale (1 point = Strongly disagree, 7 points = Strongly agree). Overall, higher scores indicate higher levels of autonomy-supportive coaching behavior.

To measure controlling behavior by a coach, a Korean version of the Controlling Coach Behaviors Scale (CCBS) developed by Bartholomw et al. [40] was developed and validated by Song and Cheon [38]. The tool consists of 15 items: four on controlling use of rewards, four on negative conditional regard, four on intimidation, and three items on excessive personal control. The 15 items include statements such as “the coach motivates me by giving me a reward when I perform well in sport”, “the coach gives me less recognition if I disappoint him/her”, and “the coach tries to interfere with my privacy outside sport.” Respondents rate each item on a five-point Likert scale (1 point = Strongly disagree, 5 points = Strongly agree).

To measure communication, a Korean version of the Scale for Effective Communication in Team Sports-2 (SECTS-2) developed by Sullivan and Short [39] was used. The Korean version was validated by Choi, ‪Cho, and Kim [40] and assesses the communication among Korean team-sport athletes. This scale emphasizes the bi-directional nature of communication between coaches and athletes with the aim of contributing to the relational dimension by narrowing the gaps between coaches and athletes, between colleagues, and between athletes and parents. The tool consists of 15 items related to four factors that measure internal aspects of individuals, including relationships between individuals as well as verbal and non-verbal communication. Specifically, the 15 items include four on acceptance, three on distinctiveness, four on positive conflict, and four on negative conflict. Respondents rated each item on a seven-point Likert scale (1 point = Strongly disagree, 7 points = Strongly agree).

To assess coach–athlete relationships, a Korean version of the Coach–Athlete Relationship Questionnaire (CART-Q) developed by Jowett and Ntoumanis [41] was used. KrCART-Q was validated among Korean athletes and coaches by Kim and Park [42]. This instrument consists of 11 items: four on closeness, three on commitment, and four items on complementarity. The items include statements such as “I like our coach” and “I have a close relationship with the coach, and I feel comfortable when receiving coaching from our coach.” Respondents were required to rate each item of the questionnaire on a seven-point Likert scale (1 point = Strongly disagree, 7 points = Strongly agree). Higher average scores are considered indicative of higher scores for each sub-factor.

The level of athlete burnout was assessed using a Korean version of the Athlete Burnout Questionnaire (ABQ) developed by Raedeke and Smith [1]. The Korean version was developed and validated by Choi, Cho, and Eklund [3] to assess burnout in Korean athletes. The ABQ consists of 15 items: five on physical and emotional exhaustion, five on reduced sense of accomplishment, and five on sport devaluation. This instrument is a questionnaire with psychological characteristics and is most widely used to measure the burnout syndrome in groups of athletes. Respondents are required to rate each item on a five-point Likert scale (1 point = Never, 5 points = Always). According to Raedeke and Smith [1], three points or higher indicates a relatively high level of athlete burnout.

### 3.4. Data Analysis

This study used two-step approach according to Anderson and Gerbing [43]. They [43] recommended validation of the measurement model before verification of the structural model. Even if the model’s overall fit is found acceptable, it is not always possible to claim that the measurement model or the structural model is supported. The measurement model and structural model should be evaluated separately. Therefore, for this study, the construct validity of the measurement model was tested. In setting the measurement model, coaching behavior that is autonomy-supportive coaching and controlling coaching were set as independent variables; communication and coach–athlete relationship were set as mediator variables, and athlete burnout was the dependent variable. The maximum likelihood (ML) method was applied to estimate the measurement model. Regarding the model fit criteria, χ^2^, CFI, TLI, RMSEA, and SRMR index values recommended by Hair, Black, Babin, Anderson, and Tatham [44] were selected as the criteria for model fit indexes. For TLI and CFI, values of 0.90 or greater were considered acceptable, and for RMSEA and SRMR, values of 0.08 or lower were deemed acceptable [45]. The mode fit criteria were also applied to verification of the structural model. In addition, in this study, the bootstrap method was applied to verify the communication roles and the coach–athlete relationship with regard to the relationship between the independent variable and the dependent variable.

Descriptive statistics, univariate skewness, univariate kurtosis, and correlations were calculated using the Statistical Package of the Social Sciences (SPSS 24.0) (IBM, New York, NY, USA). The cut-off criteria of the univariate normality assumption were absolute values of 2 for skewness and 7 for kurtosis. Additionally, AMOS 22.0 was used to conduct the structural equation modeling (SEM) to examine the full structural model.

The validity and reliability of the measurement tools were evaluated through confirmatory factor analysis. The ML method was the model estimation method to investigate whether the items of each scale in this study responded appropriately to the participants. As a result of verifying the construct validity of autonomy-supportive behavior, controlling behavior, communication, the coach–athlete relationship, and athlete burnout, some items were deleted because they did not show unidimensionality. Deleted were one item on positive conflict and two items on reduced sense of accomplishment. After the items were deleted, the model fit of each measurement tool was found to be acceptable.

Specifically, the standardized coefficient for each item about autonomy-supportive coaching behavior was 0.89–0.91, and the internal consistency (Cronbach’s α) was 0.93. The model fit indexes were found to be acceptable levels, with χ^2^ = 18.49, df = 7, TLI = 0.98, CFI = 0.99, RMSEA = 0.06, and SRMR = 0.01. The standardized coefficient for each sub-factor item for controlling behavior was 0.60–0.95, and the internal consistency values were 0.90 for controlling use of rewards, 0.89 for negative conditional regard, 0.90 for threatening and intimation, and 0.857 for excessive personal control. The model fit was relatively acceptable, with χ^2^ = 294.43, df = 81, TLI = 0.93, CFI = 0.94, RMSEA = 0.08, and SRMR = 0.06. On the other hand, the standardized coefficient for each communication sub-factor item was 0.50–0.79, and the values of internal consistency reliability were 0.79 for acceptance, 0.63 for distinctiveness, 0.65 for negative conflict, and 0.70 for positive conflict. The model fit indexes were at acceptable levels, with χ^2^ = 173.22, df = 66, TLI = 0.90, CFI = 0.93, RMSEA = 0.06, and SRMR = 0.06. In addition, the standardized coefficient of each sub-factor item of the coach–athlete relationship was 0.87-.95, and the values for internal consistency reliability were 0.95 for closeness, 0.91 for commitment, and 0.94 for complementarity. The model fit was acceptable, with χ^2^ = 119.57, df = 36, TLI = 0.97, CFI = 0.98, RMSEA = 0.08, and SRMR = 0.02.

In addition, the standardized coefficient for each sub-factor item for athlete burnout was 0.64–0.86, and the internal consistency values were 0.81 for reduced sense of accomplishment, 0.88 for emotional/physical exhaustion, and 0.89 for sport devaluation. The model fit was acceptable, with χ^2^ = 189.71, df = 61, TLI = 0.93, CFI = 0.94, RMSEA = 0.07, and SRMR = 0.04. Therefore, the validity and reliability of the scales used in this study were verified.

## 4. Results

### 4.1. Descriptive Statistics and Analysis of Correlations between Subfactors

Descriptive statistics, such as the mean and standard deviation, of the final data selected through evaluation of the measurement model were calculated (Table 2). Kline [46] suggested that if the value of skewness does not exceed an absolute value of 3, and if the value of kurtosis does not exceed an absolute value of 7, a normal distribution of data can be assumed. Based on the criteria, the assumption of normality in the data was satisfied, because the skewness and kurtosis values of each factor were within acceptable ranges for a normal distribution of data based on the reference values (skewness ≥ 0.10, kurtosis ≥ 0.20). In addition, correlation estimation showed that autonomy-supportive coaching was negatively correlated with controlling coaching (r = −0.37), while it was positively associated with communication (r = 0.53) and the coach–athlete relationship (r = 0.74). However, it was negatively correlated with athlete burnout (r = −0.60). On the other hand, controlling coaching was negatively correlated with communication (r = −0.30) and the coach–athlete relationship (r = −0.36) but was positively correlated with athlete burnout (r = 0.58). Communication had a positive correlation with the coach–athlete relationship (r = 0.55) but showed a negative correlation with athlete burnout (r = −0.48), and the coach–athlete relationship showed a negative correlation with athlete burnout (r = −0.60). With respect to the threshold at which correlation coefficients indicate statistical significance, Kline [47] suggested a threshold of 0.85.

### 4.2. Evaluation of the Measurement Model

For the measurement model (comprising five latent variables and 20 observed variables) postulated in this study, evaluations of convergent validity and discriminant validity were made (Table 3). First, in setting the measurement model, the autonomy-supportive coaching factor was posited to explain the measurement model with a latent variable. However, for controlling coaching, for communication, for the coach–athlete relationship, and for athlete burnout factors, item parceling was conducted that focused on the sub-factors presented in previous studies. This was due to the fact that if there are many measurement items, the complexity of the model increases, which can result in problems from the sample size, the model fit, and from significance tests for parametric estimation. Therefore, item parceling was carried out based on the argument by Kline [47] that if all sub-factors are set as latent variables, parsimony of the model may be violated.

This study did not use the method of deleting items in order to improve the goodness of fit in the measurement model but considered a method of using a modification index [48], and thus, correlations between error terms were assumed: two correlations within the latent variable, autonomy-supportive coaching (1↔2, 5↔6), one correlation within controlling coaching (threatening/intimidation ↔ excessive personal control), and one correlation within communication (distinctiveness ↔ positive conflict). As a result, overall, the standardized factor loading (0.6 or higher, but 0.55 for distinctiveness) and the model fit were found to be acceptable (χ^2^ = 527.13, df = 156, *p* < 0.000, TLI = 0.91, CFI = 0.92, and RMSEA = 0.08).

Table 2 and Table 3 show the construct reliability (CR) and average variance extracted (AVE) of each latent variable, the magnitude and direction of each of the correlation coefficients between variables related to the constructs, and the square of each correlation coefficient. The CR and AVE of each latent variable were calculated with the equation proposed by Hair et al. [41]. The CR values ranged from 0.79 to 0.93, exceeding the reference value (≥0.70), and the AVE values ranged from 0.50 to 0.82, also exceeding the reference value (≥0.50), showing that there was no problem with convergent validity.

The assessment of discriminant validity also showed that there was no problem with discriminant validity (AVE > φ^2^, 95% confidence interval [φ^2^ ± 2 × standard error] ≠ 1). Therefore, it can be said that the overall validity of the measurement model in this study was established.

**Figure 1:**
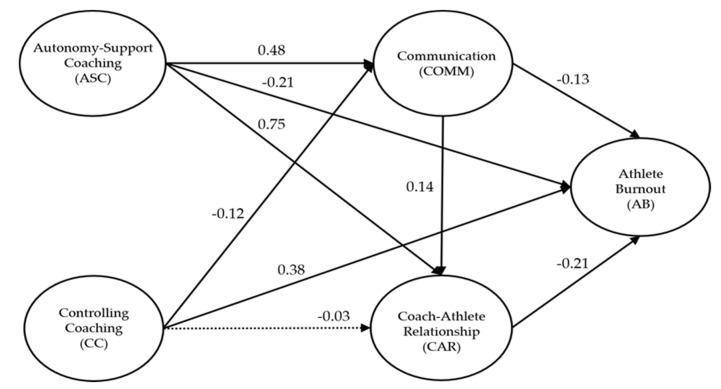
Proposed structural model. Solid lines indicate significant paths at *p* < 0.05. Dotted lines indicate insignificant paths. Values shown next to the solid lines are standardized regression coefficients.

Table 4 and Table 5 and Figure 1 show the results of analyzing the direct and indirect effects of the structural model. To determine whether to accept or reject the postulated hypotheses statistically, a statistical model to be verified by the structural model was constructed, and the structural model was verified by the maximum likelihood method according to the results from setting the statistical model. As a result of the evaluation of model fit, model fit indexes were found to be at acceptable levels (χ^2^ = 559.85, df = 156, TLI = 0.90, CFI = 0.92, and RMSEA = 0.08). Therefore, the statistical model was determined to be suitable, because the criteria for model fit indexes were satisfied overall.

As shown in Table 4, the results of analyzing the relationship of each path are summarized as follows. First, autonomy-supportive coaching was found to have a significant positive effect on communication (γ = 0.48, *p* < 0.05). Second, controlling coaching was found to have a significant negative effect on communication (γ = −0.12, *p* < 0.05). Third, communication was shown to have a significant positive effect on the coach–athlete relationship (γ = 0.14, *p* < 0.05). Fourth, autonomy-supportive coaching was found to have a significant positive effect on the coach–athlete relationship (γ = 0.75, *p* < 0.05). Fifth, controlling coaching was shown to have no significant effect on the coach–athlete relationship (γ = −0.03, *p* > 0.05). Sixth, communication was found to have a significant negative impact on athlete burnout (γ = −0.13, *p* < 0.05). Seventh, the coach–athlete relationship was found to have a significant negative effect on athlete burnout (γ = −0.21, *p* < 0.05). Eighth, autonomy-supportive coaching was found to have a significant negative effect on athlete burnout (γ = −0.21, *p* < 0.05). Finally, controlling coaching was found to have a significant positive effect on athlete burnout (γ = 0.380, *p* < 0.05).

In summary, autonomy-supportive coaching behavior had a direct positive effect on communication and the coach–athlete relationship, but it had a direct negative impact on athlete burnout. Next, communication had a direct positive effect on the coach–athlete relationship, but it had a direct negative effect on athlete burnout. As described above, results of the analysis to verify the statistical significance of each path indicated that autonomy-supportive coaching has an indirect effect on athlete burnout through communication and the coach–athlete relationship. Therefore, it is necessary to statistically verify the mediating effects of communication and the coach–athlete relationship on the relationship between autonomy-supportive coaching and athlete burnout [47].

The bootstrap method [49] was used to test the statistical significance of the indirect effects (mediation effects) of communication and the coach–athlete relationship on the relationship between autonomy-supportive coaching and athlete burnout. At this time, resampling was repeatedly conducted 5000 times, and statistical significance was assessed in the 95% bias-corrected confidence intervals (Table 5). As a result, mediating effects in the relationships of autonomy-supportive coaching→communication→coach–athlete relationship→athlete burnout (*p* < 0.05) were found to be statistically significant, and thus, it was confirmed that there was a partial mediation effect.

## 5. Discussion

In the field of sports, coaches play a significant role, fulfilling the most important position for the team as well as for the athletes. They also have a decisive effect on the overall aspects of athletes’ physical and psychological status and their performance levels [50]. In this regard, studies on the coach–athlete relationship based on the perspective of interpersonal relationships [17,51] have also suggested an efficient method for performance improvement by presenting strategies for the formation of reciprocal relationships—that is, relationships between the coach and athlete or between athletes, where people involved have favorable perceptions of each other. As a result, these studies reflect the fact that the importance of communication is emphasized in the field of sports. Therefore, this study investigated the mediation effects of communication and the coach–athlete relationship in terms of the interpersonal relationship between coaching behavior and athlete burnout.

First, autonomy-supportive coaching was found to have a positive impact on communication, but controlling coaching had a negative effect. For athletes participating in sports, the process of motivating them is very important, because the process of motivation serves as a path of action contributing to their future development, including athletic performance. Some studies have shown that teaching methods that focus on supporting autonomous motivations in students, such as their interests, needs, preferences, and personal goals, can strongly elicit students’ participatory behaviors [52,53,54]. These studies showed that autonomy-supportive coaching is required when coaches try to emphasize to athletes the goals in sports or when they try to elicit voluntary participatory behaviors (training and practice) from athletes. In addition, autonomy-supportive coaching can generate a positive relationship between the coach and athlete, and in this process, efficient communication becomes very important. It is important for horizontal and bidirectional communication—not vertical communication—to occur between the coach and the athlete. In such cases, training effects as well as the psychological satisfaction of the athletes can be increased, and horizontal and bidirectional communication can be further developed into a cooperative model in which coaches and athletes cooperate to form the training environment or atmosphere and the training effects.

In the U.S., research on the usefulness of communication in sports has been actively conducted, and communication interaction between coaches and athletes has been a major research topic [39,55,56]. Prior studies emphasized that coaches should create situations where athletes can accomplish their goals and can perform their roles efficiently. Also suggested was that it is of vital importance for coaches to not actively adhere to their positions, insist on their opinions or rights, or express their opinions strongly, but rather, they should try to behave in a way that can be perceived as considerate of the athletes. Therefore, above all, effective communication is required for interactions between the coach and athlete, and it should be preceded by the coach’s autonomy-supportive coaching.

Next, in this study, results from analysis of the relationship between coaching behavior and the coach–athlete relationship showed that autonomy-supportive coaching has a positive impact on the coach–athlete relationship, whereas controlling coaching does not influence the coach–athlete relationship. According to previous studies conducted from the self-determination theory perspective [57,58,59], the effects of autonomy-supportive coaching and controlling coaching are independent of each other. In particular, Laferniére et al. [59] reported that a coach’s autonomy-supportive coaching had a positive impact on the coach–athlete relationship (β = 0.47), whereas controlling coaching did not have any influence (β = −13). These results from Laferniére et al. [59] support the findings of the present study. In addition, these study findings suggest that research on various aspects of the coach–athlete relationships need to be continuously conducted to improve interpersonal relationships in sport situations. A consistent opinion emerging from a number of studies on coaches’ autonomy-supportive behavior is that autonomy-supportive behaviors are more likely to generate a positive relationship with coaches, because they form positive emotions in athletes. Autonomy-supportive behaviors have also been reported to contribute to building a more positive relationship and a strong emotional bond between coaches and athletes from a future-oriented point of view [60,61,62].

Considering that this study focuses on the coach–athlete relationship as described above, it is important to point out that both coaches and athletes have their respective roles to accomplish. Athletes often tend to expect the coach to lead them, give instructions to them, and make decisions for them. This is because coaches are generally presumed by athletes to be authoritative, and this fact may explain why controlling behavior is not associated with the coach–athlete relationship. In this regard, controlling behavior from coaches can be perceived as showing that they do not try to respect, care about, and understand athletes.

Therefore, in order to continuously provide useful information to athletes, there is a further need to expand the research on coaches’ autonomy-supportive behavior and controlling behavior in relation to qualitative relationships between coaches and athletes. It is believed that such research can contribute to, and lead to, the development of strategies that can further improve the relationship between the coach and athlete in the future.

On the other hand, investigation of the relationship between communication and the coach–athlete relationship revealed that communication had a positive effect on the coach–athlete relationship. This result showed that communication is an important interpersonal skill in sports and an important means for the development of the coach–athlete relationship [63]. In this regard. Liu, Chua, and Stahl [64] conceptualized the quality of communication in interpersonal relationships as a multifaceted construct that involves cognitive, behavioral, and affective elements.

Jowett and Poczwardowski [65] suggested that communication takes an important position in the coach–athlete relationship model and reported that quality and quantity in communications have a positive effect on the coach–athlete relationship. In particular, since utilization of quality communication is related to maintaining a high-level coach–athlete relationship, coaches and their athletes can share the same goals and definitions of success. Therefore, effective communication between the coach and athlete can lead to positive interactions, which will improve the performance of the athletes or increase performance satisfaction, while reducing the dropout rate or the level of burnout. Currently, in many sports, when a coach or a head coach approaches athletes for guidance or feedback to improve performance, the athletes frequently tend to avoid them and try not to make eye contact. In such sport environments, effective and efficient communication is expected to contribute greatly to increasing the effects of education, such as performance improvement and enhancing team cohesiveness. Therefore, if the coach communicates with athletes based on this value system, athletes will feel closer to the coach, and cognitive learning effects, affective learning effects, participation in training and practice, and solidarity with colleagues are all expected to increase [66].

In addition, in this study, the mediation effects of communication and the coach–athlete relationship in the relationship between coaching behavior and athlete burnout were examined. Statistical analysis indicated that communication and the coach–athlete relationship have a partial and positive mediation effect on the relationship between autonomy-supportive coaching behavior and athlete burnout. Therefore, verification of the statistical significance of each mediation effect was conducted using the bootstrap method of Shrout and Bolger [49]. As shown in Table 5, in the relationships from autonomy-supportive coaching → communication → coach–athlete relationship → athlete burnout (*p* < 0.05), the indirect effect of autonomy-supportive coaching on athlete burnout was found to be statistically significant. These results suggest that autonomy-supportive coaching increases communication with the athletes, and increased communication decreases athlete burnout by increasing the quality of the coach–athlete relationship. Therefore, it is believed that it is of the utmost importance to “keep the quality of interpersonal relationships by utilizing high-quality communication” in the field of sports. In this regard, it should be noted that maintaining effective communications in other situations, as well as training or competitions, has a positive effect on athletes’ evaluation of the communication behaviors of the coach and their own behaviors [67].

Therefore, if coaches employ effective communication skills to empathize with athletes and to meet their needs, rather than using communication to make a favorable impression on athletes, the athletes are more likely to attempt to communicate with their coaches, not only during training and competitions but also in other situations.

## 6. Conclusions

Recently, as approaches that have been applied to study the coach–athlete relationship have changed over time, the methods used by researchers to conceptualize interpersonal relationships have also changed. LaVoi [68] suggested the coach–athlete relationship is determined by each individual’s authenticity, engagement, empowerment, and ability to deal with conflict. Considering that interpersonal relationships in sports are typically unique relationships, including social interdependence, in that they are typically intention-oriented and highly outcome-oriented, if athletes can form positive relationships and interact with coaches using high-quality communication overall, they are more likely to feel that their psychological needs have been met, which will lead to a decrease in athlete burnout. Therefore, in future studies, it is necessary to verify whether the same effects of coaching behavior can be obtained among middle school and high school student athletes or professional athletes in order to reproduce the results of the present study. On the other hand, it is also necessary to investigate whether coaches use autonomy-supportive strategies more often for elite athletes or professional athletes or whether the role of coaching behavior is applied differently according to gender (male/female).

In addition, it is believed that the coach’s point of view toward mastery goals will have a significant effect on communication. A mastery goal refers to a goal to improve abilities, that is, an incremental belief, and coaches who have incremental beliefs are expected to attach value to the learning process itself; they show a tendency to continuously achieve goals, even when faced with difficulties, and tend to be interested in task maturity rather than others’ evaluations. Therefore, it is thought that the quality of communication and relationships with athletes as well as the level of athlete burnout will vary depending on the set of values pursued by the coach. It is expected that research on these issues will provide positive data that can be utilized in constructing a leader training program, including strategies for the improvement and maintenance of the relationships between leaders and athletes, and for prevention of athlete burnout in the future.

## Figures and Tables

**Table 1:** Breakdown of research participants.

Characteristics	Category	*n*	%
Sex	Male	302	87.0%
	Female	45	13.0%
Type of Sports	Golf	19	5.5%
	Basketball	79	22.7%
	Volleyball	22	6.4%
	SSireum (Korean traditional wrestling)	8	2.4%
	Baseball	82	23.6%
	Soccer	74	21.3%
	Canoe (kayak)	6	1.7%
	Table tennis	6	1.7%
	Taekwondo	8	2.4%
	Handball	43	12.4%
National team experience	Yes	85	24.5%
	No	262	75.5%
Age	19–20 years	83	23.9%
	21 years	93	26.8%
	22 years	81	23.3%
	23 years	67	19.3%
	24–29 years	23	6.7%
	Mean(age)	21.63 (SD = 1.43)	
Exercise experience	1–5 years	25	7.2%
	6–10 years	143	62.5%
	11–13 years	94	27.1%
	14 years or more	11	3.2%
	Mean (Exercise experience)	9.45 (SD = 2.42)	

**Table 2:** Descriptive statistics and correlations of scale composite scores.

Scale	M (SD)	SK	KU	1	2	3	4	5
1. Autonomy-support coaching	5.18 (1.18)	−0.21	−0.29	1				
2. Controlling coaching	3.60 (0.86)	0.21	0.64	−0.37 *	1			
3. Communication	4.43 (0.60)	−0.16	−0.13	0.53 *	−0.30 *	1		
4. Coach–athlete relationship	5.29 (1.09)	−0.21	−0.16	0.74 *	−0.36 *	0.55 *	1	
5. Athlete burnout	2.52 (0.67)	−0.09	−0.23	−0.60 *	0.58 *	−0.48 *	−0.60 *	1

* *p* < 0.05, M = mean, SD = standard deviation, SK = skewness, KU = kurtosis.

**Table 3:** Factor loading, Composite reliabilities, AVE, and Cronbach’s alpha in the Measurement model.

Latent Variable	Observed Variable	SC	CR	AVE	α
Autonomy-support coaching (ASC)	ASC1	0.85	0.93	0.69	0.95
	ASC2	0.90			
	ASC3	0.90			
	ASC4	0.88			
	ASC5	0.90			
	ASC6	0.88			
Controlling coaching (CC)	Controlling use of reward	0.63	0.80	0.51	0.86
	Negative conditional regard	0.72			
	Intimidation	0.88			
	Excessive personal control	0.84			
Communication (COMM)	Acceptance	0.96	0.79	0.50	0.67
	Distinctiveness	0.55			
	Negative conflict	0.60			
	Positive conflict	0.81			
Coach–athlete relationship (CAR)	Commitment	0.89	0.93	0.82	0.94
	Complementarity	0.95			
	Closeness	0.93			
Athlete burnout (AB)	Reduced sense of accomplishment	0.78	0.87	0.69	0.80
	Emotional and physical exhaustion	0.68			
	Sport devaluation	0.81			

SC = standardizes coefficients, CR = construct reliability, AVE = average variance extracted, α = Cronbach’s alpha.

**Table 4:** Path coefficients between Latent Variables.

Path	*b*
**Hypothesis 1**: Autonomy-supportive coaching → athlete burnout	−0.21 *
**Hypothesis 2**: Autonomy-supportive coaching → communication	0.48 *
**Hypothesis 3**: Autonomy-supportive coaching → coach–athlete relationship	0.75 *
**Hypothesis 4**: Controlling coaching → athlete burnout	0.38 *
**Hypothesis 5**: Controlling coaching → communication	−0.12 *
**Hypothesis 6**: Controlling coaching → coach–athlete relationship	−0.03
**Hypothesis 7**: Communication → athlete burnout	−0.13 *
**Hypothesis 8**: Communication → coach–athlete relationship	0.14 *
**Hypothesis 9**: Coach–athlete relationship → athlete burnout	−0.21 *

* *p* < 0.05, *b* = standardized regression weight.

**Table 5:** Estimates of mediation effect.

Path	*b*	95% CI	
		LL	UL
**Hypothesis 10**: Autonomy-supportive coaching → communication → coach–athlete relationship	0.068 *	0.023	0.142
**Hypothesis 11**: Autonomy-supportive coaching → communication → coach–athlete relationship → athlete burnout	−0.126 *	−0.233	−0.045
**Hypothesis 12**: Controlling coaching → communication → coach–athlete relationship	−0.015 *	−0.049	−0.001
**Hypothesis 13**: Controlling coaching → communication → coach–athlete relationship → athlete burnout	0.012	0.000	0.031

* *p* < 0.05, *b* = standardized regression weight, LL = lower limit, UL = upper limit, and CI = Confidence Interval.
